# Baseline atrial fibrillation is a risk factor for erectile dysfunction: Systemic review and meta-analysis

**DOI:** 10.1080/2090598X.2019.1601001

**Published:** 2019-04-24

**Authors:** Narut Prasitlumkum, Jakrin Kewcharoen, Chanavuth Kanitsoraphan, Veraprapas Kittipibul, Pakawat Chongsathidkiet, Pattara Rattanawong

**Affiliations:** a University of Hawaii Internal Medicine Residency Program, Honolulu, HI, USA; b Jackson Memorial Hospital, University of Miami Internal Medicine Program, Miami, FL, USA; c Department of Pathology, Duke University Medical Center, Durham, NC, USA; d Faculty of Medicine Ramathibodi Hospital, Mahidol University, Bangkok, Thailand

**Keywords:** Atrial fibrillation, erectile dysfunction

## Abstract

**Objective**: To assess the association between atrial fibrillation (AF) and erectile dysfunction (ED) by a systematic review of the literature and meta-analysis, as ED is commonly found amongst male patients with concurrent cardiovascular conditions, especially atherosclerosis, coronary syndrome, and diabetes; and recent studies suggest that AF is associated with ED in the general male population.

**Methods**: Studies from inception to May 2018 in the Medical Literature Analysis and Retrieval System Online (MEDLINE) and Excerpta Medica dataBASE (EMBASE) were searched. Prospective or retrospective cohort studies that compared new-onset ED between male patients with and without AF were included. Data from each study were combined using the random-effects, generic inverse variance method of DerSimonian and Laird to calculate risk ratios (RRs) and 95% confidence intervals (CIs).

**Results**: Five studies from 2007 to 2016 were included in the meta-analysis involving 29829 male patients (4096 with AF and 25733 without). The presence of AF was associated with ED (pooled RR 1.61, 95% CI 1.23–2.10; *P* < 0.001, *I*^2^ = 42%).

**Conclusions**: Baseline AF increased the risk of ED up to 1.6-fold amongst the general male population. This suggests that AF in male patients is significantly associated with ED.

**Abbreviations:** AF: atrial fibrillation; CV: cardiovascular; ED: erectile dysfunction; EMBASE: Excerpta Medica database; HR: hazard ratio; ICD-9-CM: International Classification of Diseases, Ninth Revision, Clinical Modification; (S)IR: (standardised) incidence ratio; IIEF: International Index of Erectile Function; LVDD: left ventricular diastolic dysfunction; MEDLINE: Medical Literature Analysis and Retrieval System Online; NO: nitric oxide; OR: odds ratio; RR: relative risk

## Introduction

Erectile dysfunction (ED) is defined as the consistent or recurrent inability to acquire or sustain an erection of sufficient rigidity and duration for sexual intercourse. The prevalence of ED ranges between 10% and 22% in the male population aged >20 years and is more common amongst those aged >70 years at 37% [1,2]. ED is often found in patients with cardiovascular (CV) disease or CV risk factors, such as diabetes mellitus, chronic obstructive pulmonary disease, and obstructive sleep apnoea [3,4]. In addition, several studies have shown that ED is a predictor of poor CV outcomes [5–7]. ED may be considered a surrogate marker of an occult CV condition.

Atrial fibrillation (AF) is the most common type of arrhythmia [8]. Studies have reported that AF may significantly affect sleep patterns and quality of life [9,10]. Recently, studies also have suggested that AF may be a risk factor for the development of ED [11–13]. Patients with AF have similar elevations in inflammatory markers including C-reactive protein, various types of interleukins, and endothelial/prothrombotic factors [14,15]. However, the association between AF and ED has not been clearly established. Thus, our aim in the present study was to evaluate the association between the presence of AF and incidence of ED.

## Methods

### Search strategy

**Figure 1.**
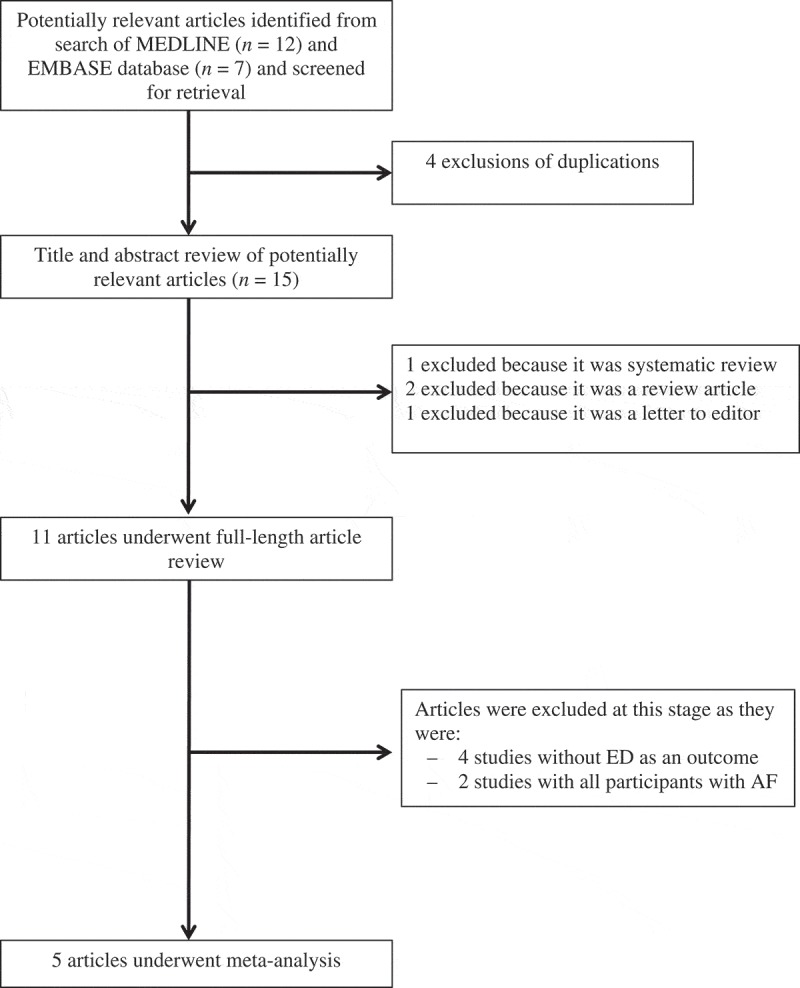
Search methodology and selection process.

Two investigators (C.K. and J.K.) independently searched indexed studies in the Medical Literature Analysis and Retrieval System Online (MEDLINE) and Excerpta Medica dataBASE (EMBASE) databases from inception to May 2018. The search strategy (Supplementary File 1) is shown in Figure 1 and included the terms: ‘atrial fibrillation’, ‘psychological sexual dysfunction’, ‘impotence’, ‘sexual disorder’, and ‘erectile dysfunction’. Only English language publications were included. The manual acquisition of other pertinent studies and review articles using references of the retrieved articles was also performed.

### Study eligibility criteria

The eligibility criteria of studies to be included were as follows:

1. Reported incidence of ED in patients with and without AF.
2. Reported relative risks (RRs), hazard ratios (HRs), odds ratios (ORs), incidence ratios (IRs), or standardised incidence ratios (SIRs) with 95% CIs (or sufficient raw data for the calculation).
3. Reported use of subjects without AF as controls.

We excluded articles that did not report RRs, HRs, ORs, IRs, or SIRs with 95% CIs and insufficient raw data for the calculation. In addition, studies with participants that either all had AF or all were without AF were excluded as there was no comparison population.

Study eligibility was independently determined by two investigators (C.K. and J.K.) and differences in eligibility were resolved by consensus. The Newcastle-Ottawa Quality Assessment Scale was used to evaluate each study in three domains: recruitment and selection of the participants, similarity and comparability between the groups, and ascertainment of the outcome of interest amongst cohort studies [16].

### Definition

ED was defined differently according to each study (Table 1 [11,13,17–19]).

**Table 1.** The clinical characteristics and summary of the included studies.

	Reference				
Variable	Chung et al., 2011 [17]	Cordero et al., 2010 [18]	Lin et al., 2015 [11]	Tokgoz et al., 2008 [19]	Yilmaz et al., 2016 [13]
Country	Taiwan	Spain	Taiwan	Turkey	Turkey
Study type	Retrospective cohort	Cross-sectional	Retrospective cohort	Cross-sectional	Cross-sectional
Participant description	Male patients seeking care for treatment of organic/psychogenic ED and male controls aged ≥40 years	Male patients with active treatment with β-blockade agents for ≥6 months	Male patients aged ≥20 years with newly diagnosed AF and age-matched controls without AF	Male patients aged 28–82 years being evaluated for ED and age-matched controls without ED	Male patients with paroxysmal lone AF and age-, gender-, and BMI-matched controls without AF
Exclusion criteria	Age <40 years, prostate/colorectal cancer	Secondary hypertension, prostate disease with or without previous surgical treatment	History of ED prior to AF diagnosis	Non-organic pathology for ED, controls with history of hypertension, diabetes, and hyperlipidaemia	Age ≥60 years, presence of confirmed risk factors for AF
No. of participants, - AF - non-AF	34 8972	147 860	3853 15,405	12 416	50 80
Age, years, mean (SD)	58.5 (11.4)	57.9 (10.6)	68.4 (13.2)	58	51.8 (7.7)
Follow-up, years, mean (SD)	N/A	N/A	4.67 (3.20)	N/A	N/A
AF definition	N/A	N/A	ICD-9-CM code 427.31	N/A	N/A
ED definition	ICD-9-CM (code 607.84 and 302.72)	Self-assessed IIEF score ≤25	ICD-9-CM code 607.84	Self-assessed IIEF score ≤21	Self-assessed IIEF score ≤21
ED prevalence, *n* (%) -AF -non-AF	6 (17.6) 1495 (16.7)	124 (84.4) 591 (68.7)	37 (0.96) 97 (0.63)	7 (58.3) 211 (50.7)	29 (58) 25 (31.2)
OR (95% CI)	N/A	2.59 (1.34–5.00)	1.53 (1.05–2.24)	N/A	1.94 (1.44–2.46)
Confounder adjustment	N/A	Age, BP control, smoking, comorbidities	Age, diabetes, hyperlipidaemia	N/A	Smoking, fasting blood glucose, uric acid
Conclusion by authors	ED is a surrogate marker for future stroke in men	AF is independently associated with ED	Presence of AF was independently associated with an increased risk of ED	Patients with organic ED are not at increased risk of ECG abnormalities.	Prevalence of ED is significantly higher in patients with paroxysmal lone AF compared with apparently healthy individuals.
Newcastle-Ottawa Quality Assessment	7	8	8	7	7

BMI, body mass index; BP, blood pressure; ECG, electrocardiogram; N/A, not applicable.

The HR was defined as an estimate of the ratio of the hazard rate in the exposed vs the control group.

### Data extraction

A standardised data collection form was used to obtain the following information from each study: title, name of first author, year of study, year of publication, country of origin, number of participants, demographic data of participants, method used to identify cases and controls, method used to diagnose the outcome of interest (ED), average duration of follow-up, adjusted and unadjusted RRs and their corresponding 95% CIs, and list of confounders that were adjusted for in multivariate analysis. To ensure accuracy, all investigators independently performed this data abstraction process. Discrepancies were resolved by referring back to the original articles and reaching a consensus.

### Statistical analysis

Meta-analysis of the combined data was performed using the random-effects, generic inverse variance method of DerSimonian and Laird [20]. The heterogeneity of the effect size estimates across these studies was quantified using the *I*^2^ statistic and *Q* statistic. For the *Q* statistic, substantial heterogeneity was defined as *P* < 0.10. The *I*^2^ statistic ranged from 0% to 100% (*I*^2^ < 25%, low heterogeneity; *I*^2^ 25–50%, moderate heterogeneity; and *I*^2^ > 50%, substantial heterogeneity) [21]. A sequential exclusion strategy, as described by Patsopoulos et al. [22], was used to examine whether overall estimates were influenced by the substantial heterogeneity observed. We sequentially and cumulatively excluded studies that accounted for the largest share of heterogeneity until *I*^2^ was <50%. We then examined whether RR estimates were consistent. In accordance with Cochrane, publication bias was assessed using a funnel plot. Funnel plot asymmetry was further confirmed with Egger’s test if there were >10 available studies [23]. All analyses were performed using STATA, version 14.1 (StataCorp., College Station, TX, USA).

## Results

### Description of included studies

The search strategy yielded 19 potentially relevant articles (seven articles from EMBASE and 12 articles from MEDLINE). After exclusion of four duplicate results, 15 articles underwent title and abstract review. At this stage, four studies were excluded because they were either a review article (two), letter to the editor (one) or systematic review (one), leaving 11 articles for detailed review. At final review, four studies were excluded for lack of ED as an outcome. Another two studies were excluded because all participants had AF and there was no control population without AF. Ultimately, five retrospective cohort studies were selected for the meta-analysis [11,13,17–19]. The pooled sample size of patients with AF was 4096 and 25733 without. The clinical characteristics are described in Table 1.

### Quality assessment of included studies

The Newcastle-Ottawa scale (0–9) was used to evaluate included studies on three domains: selection, comparability, and outcomes. Higher scores represent a higher-quality study. All studies received a score of 7 or 8, indicating high quality. Detailed evaluation of each study is presented in the Supplementary Table 1.

### Meta-analysis result

**Figure 2.**
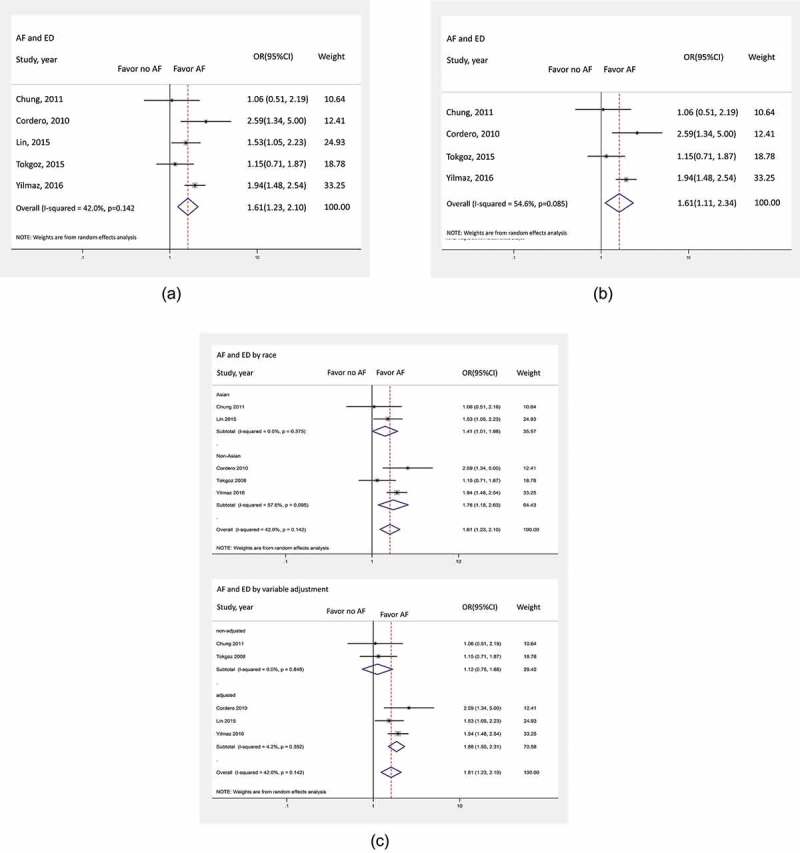
(**a**) Forest plot of studies comparing the occurrence of ED in patients with and without AF. Horizontal lines represent the 95% CIs with marker size reflecting the statistical weight of the study using the random-effects model. The diamond data marker represents the overall adjusted OR and 95% CI for the outcome of interest. (**b**) Forest plot of subgroup studies categorised by race and whether multivariable adjustment was applied, comparing the occurrence of ED in patients with and without AF. Horizontal lines represent the 95% CIs with marker size reflecting the statistical weight of the study using the random-effects model. The diamond data marker represents the overall adjusted OR and 95% CI for the outcome of interest. (**c**) Forest plot of subgroup analysis following exclusion of Cordero et al. [18] comparing the occurrence of ED in patients with and without AF. Horizontal lines represent the 95% CIs with marker size reflecting the statistical weight of the study using the random-effects model. The diamond data marker represents the overall adjusted OR and 95% CI for the outcome of interest.

Five retrospective studies with a total of 29829 participants were included in the meta-analysis. The prevalence of AF ranged from 0.3% to 38%. There was an association between AF and ED (OR 1.61, 95% CI 1.23–2.10; *P* < 0.001) with moderate heterogeneity (*I*^2^ = 42%) (Figure 2(a)). As the study by Lin et al. [11] contributed the largest number of participants, possibly dominating the analysis, it was excluded and repeat analysis was performed, which again revealed a statistical correlation between AF and ED (OR 1.61, 95% CI 1.11–2.34; *P* = 0.011) with moderate heterogeneity (*I*^2^ = 54.6%) (Figure 2(b)).

Given that the meta-analysis utilised ORs from studies with and without multivariable adjustment, we performed subgroup analyses between adjusted and unadjusted groups. This showed that AF was significantly associated with ED (OR 1.86, 95% CI 1.50–2.31; *P* < 0.001; *I*^2^ = 42%) in adjusted groups. Conversely, there was no association between AF and ED in unadjusted groups (OR 1.12, 95% CI 0.75–1.68; *P* = 0.584; *I*^2^ = 42%) (Figure 2(c)). Furthermore, race was accounted for and participants were categorised as Asian or non-Asian. The definition of ED was categorised using the International Index of Erectile Function (IIEF) score or International Classification of Diseases, Ninth Revision, Clinical Modification (ICD-9-CM) code. We found that AF, regardless of race, was significantly associated with ED. For Asians, the OR was 1.41 (95% CI 1.01–1.98; *P* = 0.044; *I*^2^ = 0%), whilst for non-Asians, the OR was 1.76 (95% CI 1.18–2.63; *P* = 0.006; *I*^2^ = 57.6%) (Figure 2(c)). Regardless of definition, both groups demonstrated a similar result: an OR of 1.76 by IIEF (95% CI 1.18–2.63; *I*^2^ = 0%; *P* = 0.006) and 1.41 by ICD-9-CM (95% CI 1.01–1.96; *I*^2^ = 0%, *P* = 0.044) (Supplementary Figure 1).

A meta-regression was performed to evaluate for a possible significant effect of ED definition, unadjusted studies, and race on the overall pooled analysis. There were non-significant changes in the risk of ED given either the IIEF or ICD-9-CM code definition (*P* = 0.117) and unadjusted studies (*P* = 0.548). Similarly, Asian or non-Asian race did not significantly affect the overall result (*P* = 0.473).

**Figure 3.**
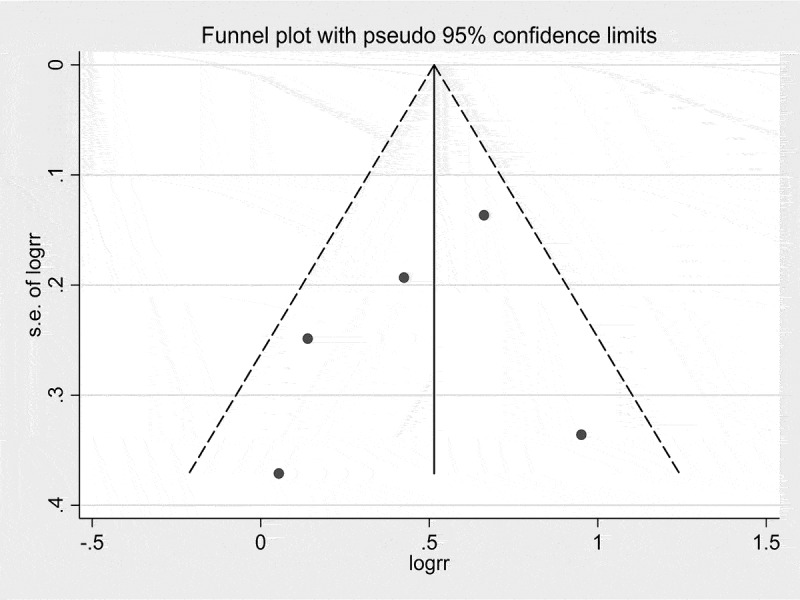
Funnel plot of AF and ED. Circles represent published studies.

Finally, the study by Cordero et al. [18] was excluded because of a relatively higher number of participants with coronary artery disease, diabetes, smokers, and β-blocker use [11,13,17,19] and the analysis was repeated (Figure 2(d)). AF remained associated with higher risk of ED (OR 1.52, 95% CI 1.15, 1.99; *P* < 0.001; *I*^2^ = 40.7%). Funnel plot analysis did not suggest publication bias (Figure 3). Egger’s test was not performed due to the low number of the studies.

## Discussion

The present meta-analysis showed an association between the presence of AF and an increased risk of ED of ~60%. The prevalence of AF in our present study ranged from 0.3% to 38%. The definition of ED used in the selected studies was either the IIEF questionnaire [24] definition or ICD-9-CM code definition. Unfortunately, only retrospective studies were included as no prospective studies were found using our search protocol.

Taking moderate heterogeneity into consideration, we conducted subgroup analyses to identify confounding variables. Two of the selected studies did not provide ORs, therefore we had to perform the calculations using the data available to include in the overall meta-analysis [17,19]. We conducted subgroup analyses based on whether each study was adjusted. We found that there was discordance between the multivariable adjusted group and unadjusted group. We believe that there were too few participants with AF in the unadjusted group [17,19] compared to studies with adjusted ORs [11,13,18], as well as no adjustment itself, leading to insufficient power to detect a difference. Further evaluation also showed that the subgroup analysis by definition of ED, either using the IIEF or ICD-9-CM code definition, was still associated with an increased risk of ED as well as race, regardless of determination. In addition, meta-regression showed no significant effect from unadjusted groups, ED definition, and race. Interestingly, in the study by Cordero et al. [18], risk of ED was higher, up to 2.6-fold, when compared to other studies [11,13,17,19]. In our view, the higher prevalence of coronary artery disease (50–72%), diabetes (28%), tobacco smoking (25%) and β-blocker administration in this study population might explain such findings. Despite the exclusion of Cordero et al. [18], the analysis nevertheless demonstrated a considerable increase in the risk of ED. The exclusion of Cordero et al. [18] may attenuate the overall impact of β-blocker use on the occurrence of ED in our present meta-analysis. In addition, given that our overall results may have been dominated by Lin et al. [11], the analysis with exclusion of this study still showed a statistical correlation, suggesting that our present analysis is robust.

Given the demographic differences between studies, particularly other medical conditions associated with ED and β-blocker use, there was noticeable heterogeneity in the present meta-analysis. These comorbidities included hypertension, hyperlipidaemia, CV, obesity, sedentary lifestyle, obstructive sleep apnoea, and tobacco smoking [25–29]. ED risk factors such as hypertension, dyslipidaemia, CV disease, obesity, sleep apnoea, and smoking are also AF risk factors [30]. This implies that AF and ED both share these risk factors and may explain the higher risk of ED in patients with AF.

The relationship between CV disease and ED has been increasingly investigated. One study [31] reported left ventricular diastolic dysfunction (LVDD) was prevalent amongst patients with ED. In addition, echocardiogram parameters including: transmitral E/A ratio, deceleration time, isovolumic relaxation time, and E/mitral E velocity/tissue Doppler imaging E velocity, were positively correlated with the severity of ED. It is theorised that generalised nitric oxide (NO) dysfunction may result in both LVDD and ED directly. In another study [32], the presence of CV disease increased the risk of ED by ~45% (OR 1.45, 95% CI 1.16–1.81; *P* < 0.01). Together with results from our present study, not only is structural heart disease, such as coronary artery disease and left ventricular dysfunction associated with ED but also, arrhythmia itself is potentially involved in ED pathophysiology [33].

The exact mechanism for the association between AF and ED has been yet to be elucidated. Several explanations have been theorised but supporting evidence remains scarce. As commonly accepted, ED is a disease of endothelial dysregulation in the penile vasculature, characterised by the interplay between inflammation, endothelial dysfunction, and hormonal disturbance [34]. In AF pathophysiology, impaired arterial distention, rising shear forces of vessels, and irregular atrial contraction may dysregulate NO production leading to ED [35]. Inflammation is related to various pathological states including oxidative stress, fibrosis, and thrombogenic processes, leading to further endothelial dysfunction and atherosclerosis. Harada et al. [36] postulated that AF is caused by inflammatory processes leading to vascular dysfunction and ultimately resulting in ED. AF could also be considered as ‘epiphenomena’, as many AF risk factors including hypertension [37]; coronary artery disease [38]; obesity [39,40]; and diabetes [41]; or the AF treatment itself, especially β-blockade [42]; may contribute to the development of ED.

### Limitations

Studies with different methodologies and demographic data were included thus forming potential sources of heterogeneity. As all studies were retrospective, the influence of residual confounders could not be completely excluded. Only five studies were included in our present meta-analysis. Thus, the funnel plot analysis may not reflect true symmetry because of insufficient power. We did not investigate whether the type of AF might impact outcomes of interest due to insufficient data. Likewise, information regarding the duration of AF in patients was not available. Thus, we could not asses the relationship between chronicity of AF and ED. The use of medication associated with ED may confound findings, especially, β-blockers and spironolactone. This question would benefit from more in-depth investigation. We excluded Cordero et al. [18] in an attempt to attenuate this effect and found that AF was still significantly associated with the risk of ED. In addition, the ICD-9-CM code definition was used in some studies to define ED, which may result in either over or underestimation of its prevalence. Not all selected studies considered ED as a primary outcome. Accordingly, our present meta-analysis was affected by study designs susceptible to type I error, outcome reporting bias, and observational bias. Finally, both adjusted and unadjusted ORs were included in our present study, which might affect the accuracy of the results. Subgroup analysis showed a discordance in our results, which could be explained by insufficient power in the unadjusted group.

## Conclusion

In summary, our present study suggests that AF may be associated with ED, warranting further study to establish the causation of AF and ED. Also, the effect of AF treatment (rate control or rhythm control) on the improvement of ED is an area of open research.

## References

[1] Rosen RC, Fisher WA, Eardley I, et al The multinational men‘s attitudes to life events and sexuality (MALES) study: I. Prevalence of erectile dysfunction and related health concerns in the general population. Curr Med Res Opin. 2004;20:607–617. 15171225 10.1185/030079904125003467

[2] Selvin E, Burnett AL, Platz EA. Prevalence and risk factors for erectile dysfunction in the US. Am J Med. 2007;120:151–157. 17275456 10.1016/j.amjmed.2006.06.010

[3] Burchardt M, Burchardt T, Baer L, et al Hypertension is associated with severe erectile dysfunction. J Urol. 2000;164:1188–1191. 10992363

[4] Phe V, Roupret M Erectile Dysfunction and diabetes: a review of the current evidence-based medicine and a synthesis of the main available therapies. Diabetes Metab. 2012;38:1–13. 22056307 10.1016/j.diabet.2011.09.003

[5] Gandaglia G, Briganti A, Jackson G, et al A systematic review of the association between erectile dysfunction and cardiovascular disease. Eur Urol. 2014;65:968–978. 24011423 10.1016/j.eururo.2013.08.023

[6] Puchalski B, Szymanski FM, Kowalik R, et al The prevalence of sexual dysfunction before myocardial infarction in population of polish men: a retrospective pilot study. Kardiol Pol. 2013;71:1168–1173. 24297714 10.5603/KP.2013.0297

[7] Shamloul R, Ghanem H Erectile dysfunction. Lancet. 2013;381:153–165. 23040455 10.1016/S0140-6736(12)60520-0

[8] Go AS, Hylek EM, Phillips KA, et al Prevalence of diagnosed atrial fibrillation in adults: national implications for rhythm management and stroke prevention: the anticoagulation and risk factors in atrial fibrillation (ATRIA) study. JAMA. 2001;285:2370–2375. 11343485 10.1001/jama.285.18.2370

[9] Szymanski FM, Filipiak KJ, Karpinski G, et al Occurrence of poor sleep quality in atrial fibrillation patients according to the EHRA score. Acta Cardiol. 2014;69:291–296. 25029874 10.1080/ac.69.3.3027832

[10] Thrall G, Lane D, Carroll D, et al Quality of life in patients with atrial fibrillation: a systematic review. Am J Med. 2006;119:448.e1-19. 10.1016/j.amjmed.2005.10.057 16651058

[11] Lin WY, Lin CS, Lin CL, et al Atrial fibrillation is associated with increased risk of erectile dysfunction: a nationwide population-based cohort study. Int J Cardiol. 2015;190:106–110. 25918058 10.1016/j.ijcard.2015.04.108

[12] Szymanski FM, Puchalski B, Filipiak KJ Obstructive sleep apnea, atrial fibrillation, and erectile dysfunction: are they only coexisting conditions or a new clinical syndrome? The concept of the OSAFED syndrome. Pol Arch Med Wewn. 2013;123:701–707. 24104459 10.20452/pamw.2017

[13] Yilmaz S, Kuyumcu MS, Akboga MK, et al The relationship between erectile dysfunction and paroxysmal lone atrial fibrillation. J Interv Card Electrophysiol. 2016;46:245–251. 26883851 10.1007/s10840-016-0115-8

[14] Guo Y, Lip GY, Apostolakis S Inflammation in atrial fibrillation. J Am Coll Cardiol. 2012;60:2263–2270. 23194937 10.1016/j.jacc.2012.04.063

[15] Vlachopoulos C, Aznaouridis K, Ioakeimidis N, et al Unfavourable endothelial and inflammatory state in erectile dysfunction patients with or without coronary artery disease. Eur Heart J. 2006;27:2640–2648. 17056702 10.1093/eurheartj/ehl341

[16] Stang A Critical evaluation of the Newcastle-Ottawa scale for the assessment of the quality of nonrandomized studies in meta-analyses. Eur J Epidemiol. 2010;25:603–605. 20652370 10.1007/s10654-010-9491-z

[17] Chung SD, Chen YK, Lin HC, et al Increased risk of stroke among men with erectile dysfunction: a nationwide population-based study. J Sex Med. 2011;8:240–246. 20722781 10.1111/j.1743-6109.2010.01973.x

[18] Cordero A, Bertomeu-Martinez V, Mazon P, et al Erectile dysfunction in high-risk hypertensive patients treated with beta-blockade agents. Cardiovasc Ther. 2010;28:15–22. 20074255 10.1111/j.1755-5922.2009.00123.x

[19] Tokgoz H, Yaman O, Gulec S, et al Electrocardiographic abnormalities in patients with organic erectile dysfunction: comparison with an age-matched control group. Int Urol Nephrol. 2008;40:373–376. 17965953 10.1007/s11255-007-9263-1

[20] DerSimonian R, Laird N Meta-analysis in clinical trials. Control Clin Trials. 1986;7:177–188. 3802833 10.1016/0197-2456(86)90046-2

[21] Higgins JP, Thompson SG, Deeks JJ, et al Measuring inconsistency in meta-analyses. Bmj. 2003;327:557–560. 12958120 10.1136/bmj.327.7414.557 PMC192859

[22] Patsopoulos NA, Evangelou E, Ioannidis JP Sensitivity of between-Study heterogeneity in meta-analysis: proposed metrics and empirical evaluation. Int J Epidemiol. 2008;37:1148–1157. 18424475 10.1093/ije/dyn065 PMC6281381

[23] Sterne JA, Egger M Funnel plots for detecting bias in meta-analysis: guidelines on choice of axis. J Clin Epidemiol. 2001;54:1046–1055. 11576817 10.1016/s0895-4356(01)00377-8

[24] Cappelleri JC, Rosen RC The sexual health inventory for men (SHIM): a 5-year review of research and clinical experience. Int J Impot Res. 2005;17:307–319. 15875061 10.1038/sj.ijir.3901327

[25] Bacon CG, Mittleman MA, Kawachi I, et al Sexual function in men older than 50 years of age: results from the health professionals follow-up study. Ann Intern Med. 2003;139:161–168. 12899583 10.7326/0003-4819-139-3-200308050-00005

[26] Budweiser S, Enderlein S, Jorres RA, et al Sleep apnea is an independent correlate of erectile and sexual dysfunction. J Sex Med. 2009;6:3147–3157. 19570042 10.1111/j.1743-6109.2009.01372.x

[27] Feldman HA, Goldstein I, Hatzichristou DG, et al Impotence and its medical and psychosocial correlates: results of the massachusetts male aging study. J Urol. 1994;151:54–61. 8254833 10.1016/s0022-5347(17)34871-1

[28] Fung MM, Bettencourt R, Barrett-Connor E Heart disease risk factors predict erectile dysfunction 25 years later: the rancho bernardo study. J Am Coll Cardiol. 2004;43:1405–1411. 15093875 10.1016/j.jacc.2003.11.041

[29] Saigal CS, Wessells H, Pace J, et al Project urologic diseases in america. Predictors and prevalence of erectile dysfunction in a racially diverse population. Arch Intern Med. 2006;166:207–212. 16432090 10.1001/archinte.166.2.207

[30] Lau DH, Nattel S, Kalman JM, et al Modifiable risk factors and atrial fibrillation. Circulation. 2017;136:583–596. 28784826 10.1161/CIRCULATIONAHA.116.023163

[31] El-Sakka AI, Morsy AM, Fagih BI Severity of erectile dysfunction could predict left ventricular diastolic dysfunction in patients without overt cardiac complaint. J Sex Med. 2011;8:2590–2597. 21679302 10.1111/j.1743-6109.2011.02350.x

[32] Grover SA, Lowensteyn I, Kaouache M, et al The prevalence of erectile dysfunction in the primary care setting: importance of risk factors for diabetes and vascular disease. Arch Intern Med. 2006;166:213–219. 16432091 10.1001/archinte.166.2.213

[33] Vlachopoulos CV, Terentes-Printzios DG, Ioakeimidis NK, et al Prediction of cardiovascular events and all-cause mortality with erectile dysfunction: a systematic review and meta-analysis of cohort studies. Circ Cardiovasc Qual Outcomes. 2013;6:99–109. 23300267 10.1161/CIRCOUTCOMES.112.966903

[34] Vlachopoulos C, Ioakeimidis N, Terentes-Printzios D, et al The triad: erectile dysfunction–endothelial dysfunction–cardiovascular disease. Curr Pharm Des. 2008;14:3700–3714. 19128223 10.2174/138161208786898716

[35] Cai H, Li Z, Goette A, et al Downregulation of endocardial nitric oxide synthase expression and nitric oxide production in atrial fibrillation: potential mechanisms for atrial thrombosis and stroke. Circulation. 2002;106:2854–2858. 12451014 10.1161/01.cir.0000039327.11661.16

[36] Harada M, Van Wagoner DR, Nattel S Role of inflammation in atrial fibrillation pathophysiology and management. Circ J. 2015;79:495–502. 25746525 10.1253/circj.CJ-15-0138 PMC4457364

[37] Krahn AD, Manfreda J, Tate RB, et al The natural history of atrial fibrillation: incidence, risk factors, and prognosis in the Manitoba Follow-up Study. Am J Med. 1995;98:476–484. 7733127 10.1016/S0002-9343(99)80348-9

[38] Crenshaw BS, Ward SR, Granger CB, et al Atrial fibrillation in the setting of acute myocardial infarction: the GUSTO-I experience. Global utilization of Streptokinase and TPA for Occluded coronary arteries. J Am Coll Cardiol. 1997;30:406–413. 9247512 10.1016/s0735-1097(97)00194-0

[39] Mahajan R, Lau DH, Brooks AG, et al Electrophysiological, electroanatomical, and structural remodeling of the atria as consequences of sustained obesity. J Am Coll Cardiol. 2015;66:1–11. 26139051 10.1016/j.jacc.2015.04.058

[40] Nalliah CJ, Sanders P, Kottkamp H, et al The role of obesity in atrial fibrillation. Eur Heart J. 2016;37:1565–1572. 26371114 10.1093/eurheartj/ehv486

[41] Benjamin EJ, Levy D, Vaziri SM, et al Independent risk factors for atrial fibrillation in a population-based cohort. The framingham heart study. JAMA. 1994;271:840–844. 8114238

[42] Ko DT, Hebert PR, Coffey CS, et al Beta-blocker therapy and symptoms of depression, fatigue, and sexual dysfunction. JAMA. 2002;288:351–357. 12117400 10.1001/jama.288.3.351

