# Supplementary material for: Baseline atrial fibrillation is a risk factor for erectile dysfunction: Systemic review and meta-analysis
Source: Arab J Urol. 2019 Apr 24;17(2):98–105. doi: 10.1080/2090598X.2019.1601001 (PMC6600068; doi:10.1080/2090598X.2019.1601001)
Supplement: Supplementary Table 1 [file TAJU_A_1601001_SM0988.docx]

**Supplementary Table 1.** Newcastle-Ottawa Quality Assessment Scale of studies included in the meta-analysis.

| Study | Selection | | | | Comparability | Outcome | | | Total |
| --- | --- | --- | --- | --- | --- | --- | --- | --- | --- |
|  | Representative of exposed cohort | Selection of the non-exposed cohort | Ascertainment of exposure | Endpoint not present at start | Comparability (Confounding) | Assessment of outcome | Follow-up duration | Adequacy follow-up |  |
| Chung et al. | * | * | * |  | * | * | * | * | 7 |
| Cordero et al. | * | * | * |  | ** | * | * | * | 8 |
| Lin et al. | * | * | * |  | ** | * | * | * | 8 |
| Tokgoz et al. | * | * | * |  | * | * | * | * | 7 |
| Yilmaz et al. | * | * | * |  | * | * | * | * | 7 |

Notes: The Newcastle-Ottawa scale uses a star system (0 to 9) to evaluate included studies on three domains: selection, comparability, and outcomes. Star (*) = item presents. Maximum 1 star (*) for selection and outcome components and 2 stars (**) for comparability components. Higher scores represent higher study quality.
