# Supplementary material for: Baseline atrial fibrillation is a risk factor for erectile dysfunction: Systemic review and meta-analysis
Source: Arab J Urol. 2019 Apr 24;17(2):98–105. doi: 10.1080/2090598X.2019.1601001 (PMC6600068; doi:10.1080/2090598X.2019.1601001)
Supplement: Supplementary Figure 1 [file TAJU_A_1601001_SM0987.docx]

**Supplementary Figure 1.**


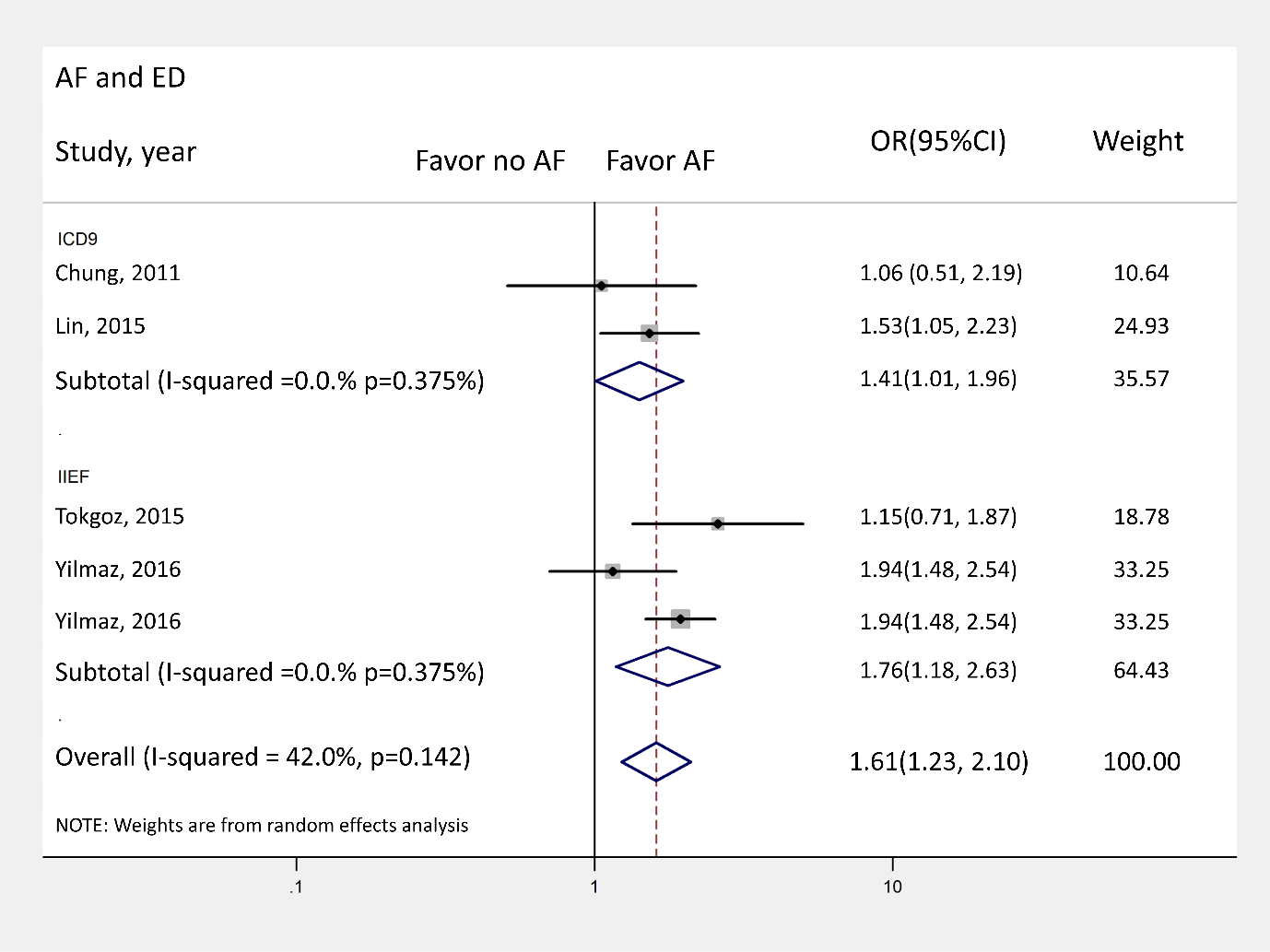


Forest plot of subgroup studies categorised by definition (either IIEF or ICD-9-CM), comparing the occurrence of ED in patients with and without AF. Horizontal lines represent the 95% CIs with marker size reflecting the statistical weight of the study using random-effects model. A diamond data marker represents the overall adjusted OR and 95% CI for the outcome of interest.
