# Supplementary material for: Baseline atrial fibrillation is a risk factor for erectile dysfunction: Systemic review and meta-analysis
Source: Arab J Urol. 2019 Apr 24;17(2):98–105. doi: 10.1080/2090598X.2019.1601001 (PMC6600068; doi:10.1080/2090598X.2019.1601001)
Supplement: Supplementary File 1 [file TAJU_A_1601001_SM0986.docx]

**Supplementary File 1. Search term and strategy.**

**EMBASE**

**Searching term**:

‘atrial fibrillation’ AND ‘erectile dysfunction’ AND male AND sexual AND [humans]/lim AND [english]/lim AND [clinical study]/lim

**Pubmed**

**Searching term:**

("atrial fibrillation"[MeSH Terms] OR ("atrial"[All Fields] AND "fibrillation"[All Fields]) OR "atrial fibrillation"[All Fields]) AND (("sexual dysfunctions, psychological"[MeSH Terms] OR ("sexual"[All Fields] AND "dysfunctions"[All Fields] AND "psychological"[All Fields]) OR "psychological sexual dysfunctions"[All Fields] OR ("psychological"[All Fields] AND "sexual"[All Fields] AND "dysfunction"[All Fields]) OR "psychological sexual dysfunction"[All Fields]) OR ("erectile dysfunction"[MeSH Terms] OR ("erectile"[All Fields] AND "dysfunction"[All Fields]) OR "erectile dysfunction"[All Fields] OR "impotence"[All Fields]) OR ("sexual dysfunction, physiological"[MeSH Terms] OR ("sexual"[All Fields] AND "dysfunction"[All Fields] AND "physiological"[All Fields]) OR "physiological sexual dysfunction"[All Fields] OR ("sex"[All Fields] AND "disorders"[All Fields]) OR "sex disorders"[All Fields]))
